# Exploring Stress and Stress-Reduction With Caregivers and Clinicians in the Neonatal Intensive Care Unit to Inform Intervention Development: Qualitative Interview Study

**DOI:** 10.2196/66401

**Published:** 2025-04-02

**Authors:** Kristin Harrison Ginsberg, Jane Alsweiler, Jenny Rogers, Phoebe Ross, Anna Serlachius

**Affiliations:** 1 Department of Psychological Medicine, Faculty of Medical and Health Sciences, University of Auckland, Private Bag 92019, Auckland, 1142, New Zealand, 64 (09) 923 5025; 2 Department of Paediatrics: Child and Youth Health, Faculty of Medical and Health Sciences, University of Auckland, Auckland, New Zealand; 3 Liggins Institute, University of Auckland, Auckland, New Zealand; 4 Department of Medicine, Faculty of Medical and Health Sciences, University of Auckland, Auckland, New Zealand

**Keywords:** neonatal intensive care unit, NICU, parents, preterm infants, stress, stress reduction, intervention development, digital, neonatology, pediatric, infants, babies, neonatal, toddler, children, caregiver, telemedicine, telehealth, virtual care, virtual health, virtual medicine, remote consultation, qualitative study

## Abstract

**Background:**

Parents and caregivers with preterm babies in the neonatal intensive care unit (NICU) experience high levels of distress and are at an increased risk of anxiety, depression, and acute stress disorders. Effective interventions to reduce this distress are well described in the literature, but this research has been conducted primarily in Europe and North America. To our knowledge, few interventions of this sort have been developed in Australasia, and none have been developed or tested in Aotearoa New Zealand.

**Objective:**

The primary aims of this study were to explore sources of stress with caregivers and clinicians in a NICU in Aotearoa New Zealand and gather participant ideas on ways to reduce caregiver stress to inform intervention development.

**Methods:**

This qualitative design used an essentialist and realist methodology to generate findings aimed at future intervention development. Overall, 10 NICU clinicians (neonatologists, nurses, and mental health clinicians) and 13 caregivers (mothers, fathers, and extended family members) of preterm babies, either currently admitted or discharged from the NICU within the last 12 months, were recruited to participate in interviews exploring stress and stress-reduction in the NICU.

**Results:**

The 23 participants included 10 clinicians (all female, with an average of 15 years of experience in the NICU) and 13 parents and caregivers (majority of them were female; 10/13, 77%) of preterm babies. We identified 6 themes relevant to intervention development. Three themes focused on caregiver stress: the emotional “rollercoaster” of NICU; lack of support, both culturally and emotionally; and caregivers feeling “left out” and confused. Three themes focused on participant-proposed solutions to reduce stress: caregiver empowerment, improving emotional support, and communication on “my” terms (ie, digitally).

**Conclusions:**

Participants reported high levels of caregiver stress in the NICU, and they proposed a range of stress-reducing solutions, including increasing caregiver empowerment and improving emotional and cultural support. Clinicians and caregivers also strongly agreed on providing more information for caregivers in digital, mobile-friendly formats.

## Introduction

Parents with babies in the neonatal intensive care unit (NICU) have described the NICU experience as “terrifying” and “traumatic” [1], and they frequently experience high rates of stress, anxiety, depression, and acute stress disorders [2,3]. These effects can last well after discharge, with long-term negative effects including problems with parent-child bonding and attachment [4].

The most common cause of admission to the NICU is preterm birth (born at less than 37 weeks gestational age). Preterm birth affects an estimated 1 in 10 births annually around the world and is the leading cause of death for children under 5 years of age [5]. In Aotearoa New Zealand, a diverse, bicultural country, preterm birth is estimated to occur in 8.9% of annual births [6]. New Zealand Europeans experience fewer preterm births, perinatal deaths, and maternal deaths than Māori, the Indigenous people of Aotearoa New Zealand who make up 17.3% of the population [7]. They also experience fewer adverse perinatal outcomes than Pacific Peoples and Indian minority groups in Aotearoa New Zealand [8]. Additionally, New Zealand European babies are less likely to be admitted to NICU than these 3 groups [9].

Effective interventions to reduce the distress of parents with babies in the NICU are well described in the literature, but this research has been conducted primarily in Europe and North America, and mostly with Caucasian mothers [10,11]. To our knowledge, few interventions of this sort have been developed in Australasia, and none have been developed or tested in Aotearoa New Zealand. As a recent study of services showed, psychosocial support for caregivers in the NICUs in Aotearoa New Zealand is highly limited, with no formalized support programs, limited cultural support services, and fewer staff members available to provide support to parents with babies in the NICU [12]. Given the limited resources available to support this highly distressed population, clinically feasible and culturally appropriate interventions for use in the NICU should be developed for the Aotearoa New Zealand context.

A few qualitative studies have explored the experiences of families in Aotearoa New Zealand NICUs [13,14], including for Māori whānau (families) [15]. However, no intervention research focused on reducing distress has been conducted with caregivers in the NICUs of Aotearoa New Zealand.

Therefore, the main objectives of this study were to gather feedback from caregivers and clinicians in the NICU on sources of caregiver stress and ways to reduce that stress to inform future intervention development.

## Methods

### Study Design

We conducted a qualitative study using semistructured interviews with caregivers and clinicians from September 2022 to April 2023 in a level 3 NICU in Auckland, Aotearoa New Zealand. We analyzed the data using thematic analysis (framework method).

### Ethical Considerations

The Consolidated Criteria for Reporting Qualitative Research (COREQ) checklist was followed to report the qualitative study findings. The Auckland Health Research Committee provided ethical consent (#AH24458). Informed written consent was provided by participants in advance of their participation in this study. Individuals were provided with a NZ $50 (US $28) gift voucher to a local store as thanks for their participation. To ensure confidentiality, all identifying details were removed from the data, and participant transcripts were issued numeric codes.

### Setting

This study was conducted at Te Toka Tumai Auckland Starship Child Health NICU, a level 3 NICU and one of Aotearoa New Zealand’s largest NICUs. Most rooms are shared, with 40 cots in the unit and an average of 900 infant admissions per year. The unit is a regional specialty center with a diverse patient population. In 2023, 29% of babies in the unit were New Zealand European, 19% were Other Asian, 17% were Indian, 17% were Pacific Peoples, and 16% were Māori. Common causes of admission were preterm birth (42%), respiratory distress (25%), and congenital abnormalities (14%) [16].

All participants in our study had admissions in the NICU during the COVID-19 pandemic. COVID-19–related visitor restrictions in the unit varied during this study’s time period, with parents recruited earlier in the study experiencing more restrictive conditions than those recruited later in the study. NICU visitor restrictions included limiting visitors in the NICU to one parent at a time, not allowing extended family visitors without special permission, and requiring all visitors to wear facial masks in the unit.

### Participants and Recruitment

We sought to collect a range of perspectives on caregiver stress and support in the NICU, so we recruited both currently admitted and discharged parents and legal guardians of preterm babies in the NICU. We also aimed to include other types of caregivers in this study, such as extended family members who had spent significant time in the NICU. This is important in the Aotearoa New Zealand cultural context, as grandparents and other extended family members (such as aunts and uncles) often play important caregiver roles in many cultures, including Māori [15].

Caregivers were eligible for the study if their baby was currently admitted to the NICU or discharged within the previous 12 months and had an admission lasting at least 2 weeks. Participants also had to be 18 years of age or older and be able to read and speak English.

Clinicians were eligible for the NICU clinician group if they were currently employed by Te Toka Tumai Auckland Starship Hospital and interacted closely with caregivers in the NICU as a standard part of their job. Eligible roles included nurses, neonatologists or pediatricians, and mental health clinicians, including social workers and psychotherapists.

An experienced research nurse in the NICU recruited parents with babies currently admitted in the unit. Parents of discharged babies were introduced to the study by clinicians using convenience sampling in an outpatient follow-up clinic for NICU graduates. Parent participants were encouraged to invite their extended family members to participate if they had spent significant time in the NICU.

Clinicians in the NICU were recruited via voluntary response sampling. Study promotion flyers for clinicians were displayed in the unit staff room and distributed by email.

Recruitment for this study was closed after data saturation was reached in both groups. Data saturation was defined as the point at which little or no relevant new categories were found in the data [17]. The final study sample size (N=23) also met the recommended sample size guidelines for achieving rigor in qualitative research of this type [18].

### Interview Schedule

The open-ended interview questions (Multimedia Appendix 1), with a caregiver version and a clinician version, were developed by the research team and based on existing literature about the sources of stress and support for parents with babies in the NICU. In the caregiver interview, part 1 included open-ended questions about sources of stress (ie, “What situations did you find most stressful in the NICU?”) and support (ie, “What helped you manage your stress most in the NICU?”). In part 2, caregivers were shown examples (websites, apps, and printed materials) of NICU education materials and evidence-based stress-reduction activities from a range of sources (both local and international) and asked for their feedback on usefulness and feasibility. Questions were a mixture of open and closed questions in part 2 (ie, “Would you have used this type of intervention in the NICU? Why or why not?”).

In the clinician interview, in part 1, clinicians were asked questions about their work with caregivers and what was most stressful and supportive from their perspective (ie, “In your experience, what types of support, education, or staff roles are most helpful in reducing stress for parents and family members?”). In part 2, clinicians were asked about their ideas on stress reduction tools and what they would like to see included (ie, “What are some features of a stress-reduction intervention that you would like to see for parents in the NICU?”). They were also asked about potential challenges involved in the implementation and feasibility of a new stress-reduction intervention.

The interview schedules were piloted with a researcher with lived NICU experience and a NICU clinician uninvolved in this study. Based on that feedback, the order of questions was changed to improve the interview flow, and some questions were simplified.

### Procedure

After expressing interest in the study, participants were contacted by a researcher who introduced them to the study and sent them a link to study enrollment materials. Participants provided informed consent and completed a demographic survey through the web-based research tool REDCap (Research Electronic Data Capture; Vanderbilt University).

Participants chose their preferred interview method (in-person or on Zoom), and families were invited to participate in interviews either individually or as a group. Each participant was given a voucher as a gift of thanks at the start of the interview.

Interviews lasted an average of 45 minutes for clinicians and 60 minutes for caregivers. Interviews were audio-recorded (if in-person) or via Zoom’s recording feature.

Two researchers conducted interviews and had no prior relationship with any of the participants. One interviewer (JR) was an experienced female Māori nurse and qualitative researcher who holds leadership roles in equity and Māori engagement. She is a mother of 5 children and grandmother of 7. The other interviewer (KHG) was a European, female, health psychology PhD candidate, licensed mental health clinician who specializes in perinatal mood disorders, and mother of 2 children.

Participants who self-identified as Māori were offered the choice of being interviewed by the non-Māori or Māori researcher. The Māori researcher conducted interviews in alignment with tikanga Māori (Māori protocols).

Interviews were transcribed using automatic speech recognition tools (Zoom and Whisper.AI [19]) and checked and corrected for accuracy using audio or video recordings. To ensure confidentiality, all identifying details were removed, and participant transcripts were issued numeric codes. Participants could review transcripts if they wished within 2 weeks after completing an interview.

### Qualitative Methodology

This study’s methodology was grounded in an essentialist and realist epistemology (that a reality exists independently of researcher beliefs or interpretations) [20] and informed by existing stress theory, including the situational stress model [21], on which the validated PSS:NICU (Parental Stress Scale: Neonatal Intensive Care Unit) was developed [22]. We used the framework method of thematic analysis, a qualitative method commonly used in applied health research [23]. The framework method allows for both a deductive approach, led by existing theory and predetermined concepts organized into a “framework,” and an inductive approach, exploring new concepts based on the data [17].

### Data Analysis

Anonymized interview transcripts were imported into the qualitative data analysis software NVivo (release 1.3, Lumivero). Data were then analyzed using the five-step process of the framework method: (1) familiarization with the data, (2) creating a coding framework, (3) indexing (coding), (4) charting (sorting and grouping of coded data), and (5) mapping and interpreting (creating themes) [23].

The initial coding framework for the caregiver dataset was developed a priori from the interview schedule and existing research on NICU parental stress using a deductive approach. It was piloted on 3 transcripts by KHG and PR. During that process, codes were also added as needed in an inductive, data-driven fashion in accordance with the framework method, which allows for both inductive and deductive approaches [17]. The same method was used to create the clinician framework. All investigators discussed and agreed on the final codes and frameworks.

Working independently and using the frameworks, 2 researchers (KHG and PR) coded transcripts, charted (grouped) the data into categories, and developed initial minor and major themes. Then, they used a collaborative approach to create the final themes, working together to compare theme ideas against transcripts and reevaluate when needed.

Initially, themes were created separately for the caregiver and clinician groups. However, after both researchers identified nearly identical themes in the 2 groups independently and shared the results with the investigative team, the decision was made to merge the datasets together. This agreement between datasets also provided triangulation, which helps ensure data rigor and credibility in qualitative research [24].

The Māori researcher (JR) also reviewed themes and example quotes for cultural understanding for the Māori participant transcripts. The final themes were reviewed and refined by all coinvestigators.

## Results

### Participant Characteristics

We interviewed 23 participants, conducting 13 interviews on Zoom (including 2 with couples) and 8 in-person: at a university (n=5), in the NICU (n=1), and, for caregivers, at participants’ homes (n=2). Three babies were present during these interviews.

Thirteen caregivers (12 parents and 1 grandparent) completed interviews and experienced NICU stays with 10 infants (8 singletons and 1 set of twins). Although we aimed to recruit multiple types of caregivers (ie, parents, grandparents, aunts, and uncles) in this study, only 1 nonparent caregiver was successfully recruited. This was likely due to COVID-19–related restrictions in the unit that ran throughout the study period and only allowed nonparent or legal guardian family members into the unit with special permission (such as to support a single mother) and did not allow children (siblings). All study families had infants admitted to the NICU due to preterm birth, and the majority of babies (6/10, 60%) had stays of 8 weeks or more.

We also interviewed 10 clinicians, who were all female and who had an average of 15 years of experience in the NICU (Table 1). Seven parents completed enrollment paperwork but did not schedule interviews and were removed from the study.

**Table 1.** Participant characteristics.

Variables	Values, n (%)
Parents or family members (n=13)	
Female	10 (77)
Ethnicity	
European	9 (69)
Māori	2 (15)
Other^a^	3 (23)
Age (years)	
21‐30	3 (23)
31‐40	7 (54)
41‐50	2 (15)
51‐60	1 (8)
Education	
Completed high school	2 (15)
Completed tertiary education	11 (85)
Employment status	
Unemployed	2 (15)
Employed part-time	4 (31)
Employed full-time	2 (15)
On parental leave	5 (38)
Baby admission details (n=10)	
Reason for admission	
Premature birth	10 (100)
Admission status	
Currently admitted	4 (40)
Discharged to home	6 (60)
Discharged: total length of stay	
6-8 weeks	2 (20)
More than 8 weeks	4 (40)
Currently admitted: length of stay	
2 to less than 4 weeks	2 (20)
More than 8 weeks	2 (20)
Clinicians (n=10)	
Female	10 (100)
Ethnicity	
European	8 (80)
Māori	1 (10)
Other^a^	1 (10)
NICU^b^ role	
Registered nurse	4 (40)
Nurse specialist or practitioner	3 (30)
Mental health clinician	2 (20)
Neonatologist or pediatrician	1 (10)
Years of experience in the NICU	
1‐10	4 (40)
11‐20	2 (20)
21‐30	4 (40)

a Chinese, Indian, Korean, Samoan; participants could select multiple ethnicities, so totals may add up to more than 100%.

b NICU: neonatal intensive care unit.

### Themes

#### Stress and Support Themes

We identified 6 themes centered on stress and stress-reduction that we considered relevant to future intervention development. Three themes focused on caregiver stress: (1) the emotional “rollercoaster” caused by the NICU experience, (2) insufficient emotional and cultural support, and (3) caregivers feeling “left out” and confused. Participant-proposed solutions to reduce stress were to (1) empower caregivers through education, (2) improve emotional support, and (3) communicate on “my” terms (ie, digitally) (Textbox 1).

Textbox 1. **Themes.**

**Sources of stress*Emotional “rollercoaster”***

- Fear and anxiety
- Overwhelmed and helpless
- Grief and loss

***Left out and confused***

- Mixed messages
- Not informed about medical care
- Lack of information

***Unsupported***

- Forgotten fathers
- Limited emotional and cultural support
- Lack of empathy

**Participant-proposed solutions**

***Empower caregivers***

- Provide basics about premature birth, the neonatal intensive care unit, and common medical procedures
- Educate on how to care for premature babies

***Communicate on “my terms”***

- Deliver information digitally
- Make mobile phone-compatible
- Simplify information

***Improve emotional support***

- Peer support, including father- and cultural-specific support
- Foster online forums and support groups
- Teach stress-reduction skills

#### Theme 1: Emotional “Rollercoaster” of the NICU

Nearly all the parent participants in our study described their time in the NICU as significantly stressful and highly distressing.

*It’s an emotional rollercoaster, with lots of crying.*

[Mother #9]

Multiple participants described it as the most stressful event of their lives.

*It’s a massive test of your emotional resources, certainly the biggest of my life, and I felt like I was having an existential crisis.*

[Mother #1]

Many parents reported ongoing episodes of fear in the NICU, related to the highly medicalized NICU environment and concerns about their babies’ survival.

*It’s terrifying to be in NICU. It’s terrifying because of so many things…seeing my baby in pain and needing so much life support to keep him alive.*

[Mother #13]

Consistently, parent participants also reported feeling overwhelmed and helpless in the NICU because of the intensity of the environment and their inability to help their babies. Fathers also shared explicit feelings of helplessness.

*My partner gave birth and can express [milk] and do all that stuff, and I can’t do any of that.*

[Father #10]

Participants also reported feelings of grief and loss throughout the NICU experience. While parents whose babies died in the unit were not part of our study, 4 participants reported sharing rooms with or befriending other families whose babies died in the unit.

*I feel like being in NICU, you not only have your own experiences, which can be quite scary, but you are also forced to take on other family’s difficult experiences too.*

[Mother #1]

Some participants also felt cultural traditions were not considered after a baby’s death.

*We felt quite unstable after a baby had passed [in the same room], and another baby just turned up. For us, it’s very tapu [sacred, with complex spiritual restrictions] when someone’s passed away. So spiritually... we felt unsafe.*

[Mother #5]

#### Theme 2: Insufficient Cultural and Emotional Support

While some parents stated they felt emotionally supported by staff in the NICU, most reported that they felt their needs were secondary.

*If you weren’t dying, you weren't a priority.*

[Mother #1]

Clinicians agreed that they often do not spend much time talking to parents about their well-being.

*We don't finish a visit by saying [to parents], “How are you feeling? Are you doing ok?” We just don't have time.*

[Clinician #8]

Multiple parents also felt that some medical staff did not respond to their emotional needs in helpful ways.

*Something had gone wrong and the doctor told me about it. And then he went on to talk about other things that could go wrong [in the future]. And I was like, that was information I needed to know, but it wasn’t information I needed to know right then. Eventually, I was like, “I want you to leave because I want to get upset in private.”*

[Mother #12]

Additionally, 3 fathers participated in our study, and most reported they felt “forgotten” and treated differently than mothers.

*I felt like [some staff] didn’t expect the dad to ask questions or be involved. Gender expectations were quite out of date.*

[Father #2]

A few mothers also commented on this, expressing frustration on behalf of their babies’ fathers about a lack of consideration for them.

*I used to joke to [baby’s dad], “you’re at the bottom, no one really cares about you.” It’s terrible, actually, I mean, talk about the gender disparity.*

[Mother #1]

All the Māori and minority participants in this study reported mixed levels of cultural support in the NICU. Three parents reported speaking their first or Indigenous language to their babies in the NICU and all expressed concerns that they were making English-speaking staff uncomfortable by doing so. The Māori participants in our study also reported concerns that staff did not understand cultural practices that were important to them.

*Because I’m brown, I felt like people were thinking, “Oh, is she capable?” I also wondered if they were treating me differently because of my skin color.*

[Mother #1]

One clinician (#5) commented on unconscious bias as a barrier to appropriate Māori cultural support, which she noted is a requirement of the Te Tiriti o Waitangi (Treaty of Waitangi), New Zealand’s founding document signed by Māori and the British Crown in 1840.

*I’m always reminding staff that we work within Te Tiriti, and that’s the place we need to start from, particularly for Māori.*

[Clinician #5]

Some participants also detailed gaps in cultural support for Asian families, including limited translation services and a lack of understanding about some cultural practices.

*So we have this cultural thing that for the first 30 days after delivery we are not supposed to go out of the house, but immediately I’m traveling between NICU and home, so that’s gone out of the window. And on the days when I wasn’t going, the nurses were ringing me like, “Are you coming today?” And I was like, “I will come tomorrow.” You have this pressure on you.*

[Mother #8]

#### Theme 3: Caregivers “Left Out” and Confused

Caregivers reported significant confusion and uncertainty in the NICU due to a lack of information, which they reported increased their stress. Many reported having unanswered questions about day-to-day procedures in the NICU, who the staff were that cared for their babies, and what they could expect in the future.

*When I went into labour, I didn’t know how early you could have a baby, or what the survival rate was. I didn’t know what NICU was! I wish someone had explained it all to me more clearly.*

[Mother #6]

A few caregivers also noted that it was difficult for them to take in information early in their baby’s admission due to the suddenness of their baby’s birth and the feelings of fear and overwhelm they were experiencing.

*We’re almost three months into our admission, and I’m probably only now just getting to a point where I can mentally understand everything that happens in NICU and communicate about it clearly. It was just a real shit show for a long time, and of course I had to recover from birth. It probably took me eight weeks to feel like myself.*

[Mother #12]

Some mothers also reported feeling nervous about asking questions of the medical team.

*I know at the start, I remember sitting there and wanting to say things [to the doctors]. But even just asking something was nerve-wracking and overwhelming.*

[Mother #8]

Similarly, a few clinicians expressed the concern that the information they were providing was not always understood by caregivers, and they attributed this to both a lack of time they had available to talk to families and potential power differentials between medical providers and caregivers.

*The [medical] hierarchy is sometimes an issue for people to speak up and ask more questions. They may come back later and say, “Look, I actually didn’t understand a word.” Or they may come back and ask a nurse but not the doctor.*

[Clinician #4]

Participants also mentioned feeling more stressed when they received conflicting information from different staff members.

*[My baby] couldn’t latch properly, and the machine would start beeping [about his oxygen], and I had different messages from the nurses. So some nurses say “it’s totally fine,” and then some nurses will say, “If he drops then you need to stop breastfeeding and let him breathe.” The mixed messages gave me a lot of anxiety.*

[Mother #8]

Concerningly, many parents also reported stressful situations in which they were not informed about their babies’ medical care. One mother (#6) reported a “really scary” incident when she was not told that her baby had stopped breathing, which she reported “made me lose my mind.” Additionally, 2 participants reported being told “at the last minute” or without their knowledge that their babies were moved to a different section of the unit.

*So before we moved from Level 3 to Level 2, we were told, “It’s not going to happen for a while.” But then we’re told over the phone that now she’s in Level 2. That was very stressful for us. The doctor had given us clear plans, yet the transition happened without us.*

[Mother #5]

Clinicians also reported stressful situations when parents were “left out of the conversation,” attributing this to time constraints and frequent staff changes.

*If we had more staff, that would give me more time to talk to parents, rather than thinking, “Oh, but I’ve got these 3 other babies to think about.” If the staff is not stressed, then everybody can be less stressed.*

[Clinician #3]

#### Theme 4: Empower Parents

Overwhelmingly, participants expressed an interest in learning more about the NICU and learning how to better care for and respond to their preterm babies in the NICU. Generally, caregivers wanted information that would help them feel more confident and empowered in the NICU.

*The things at the top of my education wish-list would be on breastfeeding and pumping, which was so stressful; how to hold your baby; the importance of skin-to-skin and any other developmental tools I could use; and understanding your baby’s behaviour.*

[Mother #9]

Clinicians echoed this idea and felt it important to emphasize the importance of caregiver involvement and partnership with the medical team in the NICU.

*I think an intervention that would include solid medical information and highlight ways for parents to be involved in NICU would be really helpful. It needs to have a big focus on what parents can do, because there’s so much already taken away from them. And focus much more on the message, ‘this is your baby,’ and emphasize that they are part of the medical team.*

[Clinician #4]

Participants also had many specific ideas about the types of NICU information that would be helpful to them, as well as a desire to receive personalized information about their babies.

*I wanted to know more about everything. How long does my baby need to be on the incubator, how long for the overall stay, the timing of things. Also, their growth, their weight, are they on track?*

[Mother #8]

Some participants had specific information requests, from standard medical procedure timelines to developmental week-by-week guides.

*It would be nice to have a brief timeline of when babies will get the brain scans, when they’ll get their first eye checks. It would be good to know when things are going to happen before, instead of after, so you can ask if you should be there and know to ask about the results.*

[Mother #11]

Some parent participants also commented on the lack of information available to them about their babies’ medical care through the hospital’s electronic health record system, and one suggested an “integrated” web-based approach to include both NICU education and updates about their babies’ medical care.

*It’d be great to have education about NICU in an app form that also includes the doctor’s notes from the day. I’d like to have more information that keeps you updated when you aren’t there.*

[Mother #11]

#### Theme 5: Provide More Emotional Support

Most participants stated they wanted more emotional support, including more empathy from staff and more ways to destress. Participants also mentioned a desire to connect with other caregivers “like me,” such as fathers or caregivers from the same cultural group.

*It would have been nice to chat with a few more dads. If there was some way that brought people together in a casual format, that’d interest me.*

[Father #10]

Some participants expressed a desire to connect both in-person and via online support groups with caregivers from the same NICU.

*It’d be great if there was an online forum or group for parents from this NICU. I found connecting to other NICU parents helped online, even if their baby is no longer in NICU or was in another city. Also, if they can invite some ex-NICU parents [to the unit], I think that would be really good too.*

[Mother #8]

Clinicians also commented on the strength of support parents could provide to each other.

*It’s all very well for us as professionals to say “I’ve been here for years, and this is what 25-week-old babies do.*” *But that doesn’t make a parent feel better sometimes. But if another parent who had a 25-week-old maybe two years ago can say, “Look, now I have a healthy child,*” *that’s hugely helpful.*

[Clinician #1]

In addition to peer-support as a source of stress reduction, many caregivers also expressed an interest in learning specific stress-reduction skills that they could use while in the NICU.

*It would be quite nice to put on headphones and do some breathing exercises or to listen to a guided meditation.*

[Caregiver #7]

Clinicians expressed an interest in having more ways to help parents reduce stress.

*We know parents are really stressed, and it'd be great to be able to say, “Here are some tools to reduce your stress.”*

[Clinician #2]

A few participants also noted that there is a lot of downtime in the NICU, and having stress-reducing activities to try would be more helpful than “doom scrolling” on a phone.

*Based on my experience, I spent a lot of time in NICU just sitting there not doing anything, especially in my baby’s early days. So I would have tried some stress-reduction activities if someone had given them to me, because hopefully they’d help me feel better, but also to give me something to do.*

[Mother #9]

One mother stated that she and her partner had a session with a psychotherapist during their baby’s admission, which she reported helped reduce her stress. However, she noted that scheduling these sessions was challenging and privacy was limited within the unit. (Clinicians also noted similar challenges in this regard.) She recommended complementing this type of in-person therapy with resources and activities that could be done at home.

*The therapist gave us self-reflective questions to think about, and those were really helpful for us to talk about later. Like, what was most important to us as parents in NICU? It’s hard to get perspective when you are in the midst of it, and those reflective questions really helped us do that. I think a list of those types of questions could be really useful for other parents, too.*

[Mother #12]

Participants also recommended ways to improve cultural support, including more diverse staffing and staff training on cultural traditions.

*I think it’d be helpful to have really good information in different languages and then basic info for staff [on cultural traditions], like this is something a family might want to do, and here’s how to support them.*

[Clinician #1]

Two Māori participants also recommended specific cultural practices around stress reduction.

*I’m always interested in karakia (Māori ritual chants/incantations) and ones to do with breathing. For me, it’s really helpful in stressful situations and really important that I keep doing it in the NICU.*

[Mother #13]

#### Theme 6: Communicate on “My” Terms (ie, Digitally)

Many caregivers and clinicians expressed an interest in authoritative, reliable web-based resources for information about the NICU and premature baby development, and they commented that the printed resources currently provided in the unit were “not helpful.”

*I’ve spent more than a decade dedicated to the NICU, and I wouldn’t read our pamphlets. Also, we’re all on our phones. That’s how we communicate. So I think digital is way better.*

[Clinician #6]

Another clinician agreed, observing that younger parents in particular prefer digital information.

*Digital is the way to go. The younger parents never read a printed pamphlet. They take out their phone, and they find their answers, good or bad. I think content needs to be short, sharp, and ideally, more video rather than lots of text. I think it should also be easy to navigate so that when they are hopefully sitting at the bedside or expressing milk, they can look at it when they have a few minutes.*

[Clinician #4]

While 2 caregivers preferred printed materials over web-based, the majority of caregivers wanted information delivered in digital formats and specifically mobile-friendly.

*I never open my laptop. I’m always on my phone. You want it to be easy to read and less words and more videos. But you do want to find the right information that’s accurate for your baby.*

[Mother #12]

Three participants brought specific app and blog ideas they had brainstormed to their study interviews because they felt “so frustrated” by the lack of information they felt they received about the NICU and premature babies. They also noted they wanted to be able to access information regardless of the time of day.

*I want more information about my baby and NICU, and I want to be able to work things out in my own time, online, even if it’s at 2 am.*

[Father #2]

## Discussion

### Principal Findings

Our study explored caregiver stress in the NICU and solicited participant ideas on ways to reduce stress to inform future intervention development. Broadly, our stress themes highlight how the majority of caregivers felt devalued in the NICU and that their emotional needs were not a priority. They expressed a desire to be treated as partners in their babies’ medical care and seen as individuals, with unique values and strengths that were vital to their babies’ health and well-being.

These findings align with stress theory, which posits that stress occurs in response to specific components of a situation as well as an individual’s perceptions of that environment and their ability to cope within it [21]. The PSS:NICU [22], which was developed based on this theory, measures parental perceptions’ of stress in response to four specific components of the NICU environment: (1) “sights and sounds of the NICU,” (2) “infant behavior and appearance,” (3) “parental role alteration” (with examples such as “not being able to hold my baby” and “feeling helpless”), and (4) “staff communication and behaviors.”

Our study data contain examples of all of the categories measured by the PSS:NICU. Importantly, the majority of our data about parental stress can be organized within the category of “parental role alteration.” In particular, our themes around parents feeling left out of decisions about their babies’ medical care, uniformed, and unsupported emotionally highlight how parents felt disempowered in the NICU. This is important, as a meta-analysis of studies from around the world using the PSS:NICU found that “parental role alteration” is the most significant factor in parental stress in the NICU, with higher perceptions of changes in the parental role leading to higher rates of parental stress [25].

The stress themes in our study highlight problematic gaps in emotional and cultural support for families that led to feelings of parental isolation and disempowerment. While some parents stated they were supported in the NICU, the majority reported stressful interactions that made them feel devalued, confused, or judged as parents. Fathers, in particular, reported feeling “less important” than mothers in the NICU. Fathers’ needs have long been understudied in the NICU, with many studies focusing exclusively on mothers [5]. However, recent studies have demonstrated that fathers can experience high levels of distress in the NICU, including elevated rates of depression and posttraumatic stress disorder compared to non-NICU fathers, and specialized interventions in the NICU can help [26].

In total, 30% (7/23) of our study participants self-identified from Māori and other minority cultures. These participants all reported mixed experiences of cultural safety (such as feeling judged by speaking a non-English language to their baby in front of medical staff), which are similar to findings reported in previous studies internationally with minority families [27] and in Aotearoa New Zealand with Māori families in the NICU [15].

Cultural support is a critical area for improvement and should be considered an important component of parental empowerment in the NICU [28] and an avenue for future intervention development. As a systematic review of Indigenous populations’ birth outcomes in New Zealand, Australia, Canada, and the United States found, Indigenous groups experience significantly higher rates of preterm birth and neonatal death than non-Indigenous populations [29], and a growing body of international research has also documented a strong dose-dependent relationship between experiences of discrimination and health outcomes [30]. Few studies have explored experiences of discrimination and health outcomes within the NICU [31] or how cultural support can affect outcomes, and these are much-needed avenues of future research.

All participants shared ideas on ways to reduce parental stress in the NICU and what they felt would be helpful in a future intervention. Participants overwhelmingly agreed on the need for caregivers to feel more confident and empowered in the NICU. This idea has significant support in the literature, with decades of evidence documenting the benefits for parents and babies of increased parental involvement and engagement in the NICU [32]. As our study highlights, however, this knowledge can be difficult to translate into practice. Formalized “empowerment” interventions in the NICU have been designed to address this challenge, and most involve multimodal intervention programs that include hands-on caregiver education and changes in clinical practices. These have been found effective in reducing parental stress and depression [32].

Caregiver participants were interested in learning about education topics in 2 categories: how to care for their baby in the NICU and understanding the NICU environment, which was new and unfamiliar to all the families in our study. This desire is backed by research: educational interventions for parents with babies in the NICU have been shown to improve parenting confidence and decrease parental anxiety [33]. Clinicians echoed this need, and most participants wanted information to be provided in “short and sharp,” easy-to-understand text and video. Participants also overwhelmingly agreed on the need for digital, mobile-friendly delivery of information (both for education and about their babies).

Digital interventions are a worthwhile avenue for future exploration, as evidence has demonstrated the effectiveness of digital interventions with pregnant and postnatal women. A 2024 meta-analysis examined 31 randomized controlled trials testing digital intervention effects on postpartum anxiety and depression and found significant reductions in symptoms compared to treatment as usual, particularly for interventions that incorporated psychotherapy such as cognitive behavioral therapy or mindfulness [34]. Despite this evidence base, to our knowledge, no digital mental health interventions have been evaluated for parents with babies in the NICU.

Participants also proposed a range of strategies to improve emotional support, including through parent-to-parent support (both in-person and through online support groups). Participants also wanted improved cultural support, including increased staff training on diverse cultural traditions, hiring more Māori and minority staff, and creating more ways for parents to connect with other parents from similar cultures. This personal connection aligns with the Māori concept of whakawhanaungatanga, which emphasizes building meaningful, trusting relationships.

### Strengths, Limitations, and Future Research

To inform future intervention development, this study explored sources of stress and solicited ideas about ways to reduce stress with caregivers and clinicians in a level 3 NICU in Aotearoa New Zealand. Soliciting participants’ ideas on caregiver stress reduction in the NICU is an uncommon approach in the literature and a strength of the study.

This study is qualitative, and therefore its findings are not generalizable but may be transferable to similar populations. Our results are reflective of one participant group, in which the majority of participants were female, self-identified as European in ethnicity, and most were tertiary-level educated and employed. The study interviews were also conducted during the COVID-19 pandemic, which may have influenced participants’ perceptions of stress and contributed to their ideas about stress reduction (such as more interest in digital sources of information).

In the recruitment process, 7 parent participants completed enrollment paperwork but did not schedule interviews. We do not know the reasons for this change in participation interest; however, we were aware of possible barriers, including parental hospitalization, a transfer out of the unit, and a lack of access to a mobile phone, limiting interview scheduling.

Future studies should strive to reduce barriers to participation in research for all caregivers with babies in the NICU, and they should aim to recruit more fathers and minority participants, who are underrepresented in the literature [5]. Caregivers and clinicians in the NICU are also valuable, knowledgeable resources for intervention development and design. Involving stakeholders such as parents, extended family members, and clinicians in intervention development is likely to improve patient-centered care, improve engagement in interventions, reduce inefficiencies in research, and improve research outcomes [35].

### Conclusions

This study explored sources of parental stress in the NICU with caregivers and clinicians, and gathered participants’ ideas and feedback on ways to reduce stress to inform future intervention development. Proposed solutions by participants focused on increasing parental empowerment, improving emotional and cultural support, and providing information in digital, mobile-friendly formats. This formative study was essential in identifying the unique needs and views of both caregivers and clinicians working in the NICU. It has since informed the development of a digitally delivered psychoeducational program that we are currently evaluating in a randomized controlled trial [36].

## Supplementary material

10.2196/66401 Multimedia Appendix 1 Semistructured interview outlines.

## Abbreviations

COREQ: Consolidated Criteria for Reporting Qualitative Research
NICU: neonatal intensive care unit
PSS:NICU: Parental Stress Scale: Neonatal Intensive Care Unit
REDCap: Research Electronic Data Capture
