# Supplementary material for: Exploring Stress and Stress-Reduction With Caregivers and Clinicians in the Neonatal Intensive Care Unit to Inform Intervention Development: Qualitative Interview Study
Source: JMIR Pediatr Parent. 2025 Apr 2;8:e66401. doi: 10.2196/66401 (PMC12038759; doi:10.2196/66401)
Supplement: Multimedia Appendix 1 [file pediatrics-v8-e66401-s001.docx]

### Appendix 1: Semi-Structured Interview Outlines

**Caregiver Questionnaire**

**Part 1: NICU Experience**

1. (Extended family only: How were you involved in supporting the baby and their parents in the NICU?)
2. What words/emotions come to mind when you think about your time in the NICU?
3. How would you describe your stress levels during this time?
4. What situations did you find most stressful?
5. Did you have experiences in the NICU that felt culturally unsafe or

unsupportive? If yes, how could the situation have been handled better?

1. What support helped you most in terms of managing your stress during your time in the NICU?
2. What support or information would you have liked to receive that you didn’t receive?

**Part 2: Feedback & Ideas on Stress-Reduction Tools**

*Handouts & Booklets* (Examples: Little Miracles Trust booklet; Mother to Baby mindfulness script)

1. What do you think about this type of written information?
2. In general, do you think more handouts of this sort would have been

helpful to receive? Why or why not?

*Websites* – (Examples: COPE: Centre for Perinatal Excellence; COCOON]

1. Do you like the look of this website? Why/why not?
2. Would you use this website during your NICU stay? Why/why not?
3. What content would you find helpful on a website like this?

*Apps –* [Examples: IFDC app, ACTCompanion]

1. Do you like the look of these apps? Why/why not?
2. Would you use an app like this during your NICU stay? Why/why not?
3. What content would you find helpful on an app like this to reduce stress?

Do you have any other ideas or feedback you’d like to share that we haven’t covered?

**Clinician Questionnaire**

**Part 1: NICU Experience Supporting Parents**

1. What are your responsibilities related to supporting parents in the NICU?
2. What are the most challenging parts of these responsibilities?
3. How have COVID restrictions impacted your work with parents? Have they added additional stressors?
4. In your experience, what types of support / education / staff roles are most helpful in reducing stress for parents?
5. What support or information would you like parents to receive / have access to that they don’t currently?

**Part 2: Ideas for Stress-Reduction**

1. Would you encourage NICU parents to use an app or website for stress reduction or to develop coping skills? Why or why not?
2. What are some features of a stress-reduction intervention that you would like to see for parents in the NICU?
3. Why would these features be helpful for NICU parents?
4. What are some features of apps that you do not want to see in an intervention of this sort?
5. Why would these features be unhelpful for NICU parents?
6. What delivery method of intervention – printed materials, website, app -- do you think would be most effective for parents in the NICU? Why?
7. What challenges / problems do you foresee with delivering an intervention in the NICU to parents?

Do you have any other ideas or feedback you’d like to share that we haven’t covered?
